# People’s Voice and Civil Society Participation as a Core Element of Universal Health Coverage Reforms: Review of Experiences in Iran

**DOI:** 10.34172/ijhpm.2021.123

**Published:** 2021-09-06

**Authors:** Dheepa Rajan, Mohammad Hadi Ayazi, Maziar Moradi-Lakeh, Narges Rostamigooran, Maryam Rahbari, Behzad Damari, Ali Asghar Farshad, Reza Majdzedeh, Kira Koch

**Affiliations:** ^1^Health System Governance and Financing Department, World Health Organization (WHO), Geneva, Switzerland.; ^2^Shahr Ray Azad University, Tehran, Iran.; ^3^Preventive Medicine and Public Health Research Center, Psychosocial Health Research Institute, Iran University of Medical Sciences, Tehran, Iran.; ^4^Secretariat of Supreme Council of Health and Food Security, Ministry of Health and Health Education; Tehran, Iran.; ^5^Community Based Participatory Research Center, Iranian Institute for Reduction of High – Risk Behaviors, Tehran University of Medical Sciences, Tehran, Iran.; ^6^Department of Governance and Health, Institute of Neuroscience, Tehran University of Medical Sciences, Tehran, Iran.; ^7^Occupational Health Research Center, Iran University of Medical Sciences, Tehran, Iran.; ^8^Community Based Participatory Research Center, Knowledge Utilization Research Center, Tehran University of Medical Sciences, Tehran, Iran.

**Keywords:** Universal Health Coverage, Health System Governance, Participatory Governance, Social Participation, Health Transformation Plan, Islamic Republic of Iran

## Abstract

Health governance challenges can make or break universal health coverage (UHC) reforms. One of the biggest health governance challenges is ensuring meaningful participation and adequately reflecting people’s voice in health policies and implementation. Recognizing this, Iran’s Health Transformation Plan (HTP) lays out the country’s blueprint for UHC with an explicit emphasis on the ‘socialization of health.’ ‘Socialization’ is seen as a key means to contribute to HTP objectives, meaning the systematic and targeted engagement of the population, communities, and civil society in health sector activities. Given its specific cultural and historical context, we sought to discern what notions such as ‘civil society,’ ‘non-governmental organization,’ etc mean in practice in Iran, with the aim of offering policy options for strengthening and institutionalizing public participation in health within the context of the HTP. For this, we reviewed the literature and analysed primary qualitative data. We found that it may be more useful to understand Iranian civil society through its actions, ie, defined by its motivation and activities rather than the prevailing international development understanding of civil society as a structure which is completely independent of the state. We highlight the blurry boundaries between the different types of civil society organizations (CSOs) and government institutions and initiatives, as well as high levels of overlaps and fragmentation. Reducing fragmentation as a policy goal could help channel resources more efficiently towards common HTP objectives. The National Health Assembly (NHA) model which was first launched in 2017 offers a unique platform for this coordination role, and could be leveraged accordingly.

## Background

 There are increasing calls for universal health coverage (UHC) design to include participatory multi-stakeholder governance mechanisms, with advocates and critics pointing to health system gaps in ensuring responsiveness to the public’s needs.^1^ The current coronavirus disease 2019 (COVID-19) pandemic has directed a glaring spotlight at those gaps, with governments often making closed-door decisions with a narrow group of medical-technical experts, neglecting experiential knowledge from affected populations and civil society.^2,3^ Especially in times of crisis, ignoring people’s voices erodes the public’s trust in government^4^; thus, the very participatory mechanisms needed to build and maintain trust must be a core element of the health system modus operandi, an investment made steadily over time to fine-tune meaningful engagement with the population, communities and civil society.^3^

 Recognizing this, Iran laid out an ambitious Health Transformation Plan (HTP) in 2014 which provided the country’s blueprint for UHC reforms.^5,6^ The HTP’s stated objectives are: (*a*) to improve the stability of financial resources for health; (*b*) ensure financial protection against undue hardship from out-of-pocket expenses; and (*c*) increase access to high-quality health services. The ‘socialization of health’ is seen as one of the means to contribute to these objectives,^7^ generally meaning the systematic and targeted engagement of the population, communities, and civil society in health sector activities.^8^

 This paper is drawn from a review of the ‘socialization of health’ approach, conducted in 2017-2018 by the World Health Organization (WHO), the Iranian Ministry of Health and Medical Education (MoHME), and the National Institute for Health Research (NIHR), as part of a larger evaluation of the state of Iran’s health system governance and financing.^9^ This study focused on a sub-set of the data with the aim of gaining insight into (*a*) the real challenges for increased and effective participatory governance in the health sector and (*b*) what works well enough to scale up.

 Here, we specifically highlight the need for a more nuanced understanding of multi-faceted notions of ‘civil society’ and ‘non-governmental organization’ (NGO)^10^ in a singular cultural and historical context such as Iran.^11^ Based on that contextual understanding, we then use primary and secondary data to examine organized forms of public engagement and reflect on policy options for strengthening and institutionalizing public participation in health within the context of the HTP. Finally, we reflect on how the lessons drawn from this study can be useful for other middle-income country contexts, each with its own unique history and political system.

## Methods

###  Review Methodology 

####  Literature Review

 A literature review of published documents was undertaken in Farsi and English in 2017.

 Cochrane, Google Scholar, JSTOR, Project Muse, and PubMed were searched for English-language literature (search terms in Figure) published back to 2005. On Google Scholar, the number of hits generated were over 1000; the function ‘sort by relevant’ helped narrow down the number of hits, and the abstracts of the top 40 articles were reviewed for inclusion or exclusion. On Cochrane, the top 30 articles were screened. On JSTOR, the top 20 non-duplicate abstracts were reviewed. From PubMed, 35 non-duplicate abstracts were reviewed. On Project Muse, only a few hits were found and deemed not relevant for inclusion into the study.

**Figure:**
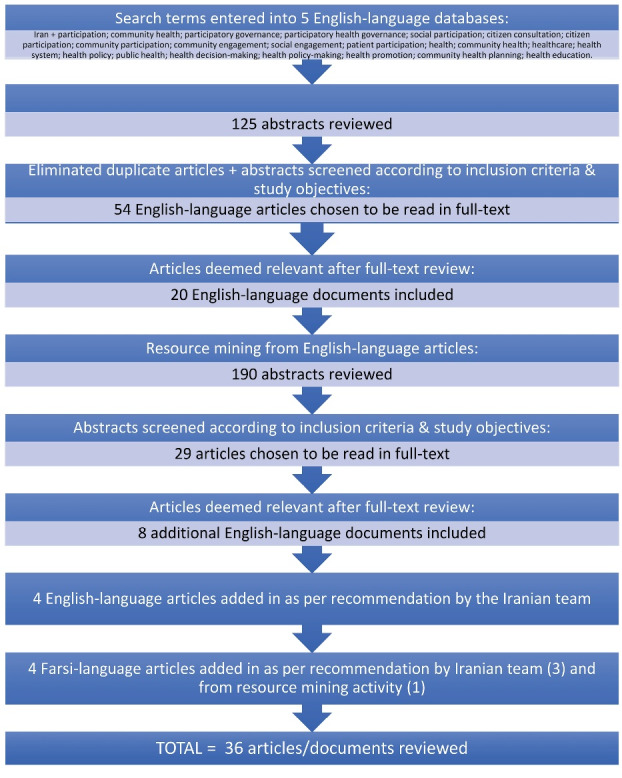
Literature Search Methodology.

 In total, 54 documents were deemed relevant for full-text review based on the following inclusion criteria: (1) the studies were in English; (2) the studies contained one or more search terms.

 The inclusion criterion was relevance with the study objectives of understanding organized forms of public engagement and participatory governance mechanisms in Iran’s health sector. Exclusion criteria were: no link to either Iran, the health sector, or participatory mechanisms (34 articles were discarded). Finally, 20 English-language articles were included into the study.

 Four additional English-language articles were recommended for inclusion by the Iranian team. Reference mining was undertaken for all English-language articles, leading to 190 further abstracts. Of these, 29 were deemed relevant for full-text review, and 8 were included into the study.

 Preliminary findings from the English-language literature were presented to a government-led health sector stakeholder group in Tehran in October 2017. Based on the feedback and ensuing discussion, a Farsi-language literature review and qualitative primary data collection was added to the study plan.

 Persian-language articles were hence accessed in health.barakatkns.com and Iran Medex. Persian search terms for ‘people,’ ‘participation,’ and ‘health’ were used (people: مردم; participation: مشارکت; health: سلامت). All 1232 hits’ article titles were reviewed for relevance with the study objectives. 65 abstracts and 29 full-text documents were reviewed, with 10 articles included. The team’s Persian speakers read the 10 full-texts, and included 3 articles. One additional Persian article was added after mining the references of the 3 included Farsi articles, bringing the total number of included articles to 36 (Figure). The study objectives were used as a framework for analysis.

####  Key Informant Interviews

 Key informant and group interviews were undertaken in February 2018 in Tehran and Qazvin provinces: 4 government representatives, 3 community-based organizations (CBOs), 2 civil society organizations (CSOs), and 2 parliamentarians. Working in Excel, a preliminary coding framework with broad common themes were derived from co-author notes from interviews, the literature review, the October 2017 stakeholder meeting discussions, and exchanges between WHO, MoHME, and NIHR.

 Interviews were transcribed into Persian. A certified translator provided English translations. Co-authors analyzed the transcripts by applying the coding framework to the transcripts and modifying and updating the framework with additional themes emerging from the data (deductive-inductive mixed approach).

 Four researchers with differing, or no, institutional affiliations (1 WHO, 1 MoHME, 1 NIHR, 1 external) independently coded the transcripts. Each transcript was coded by at least 2 out of 4 people. Every single coded text passage was reviewed by at least 3 team members through online meeting sessions where discordances and differing understandings were discussed in detail, and a consensus reached. The original Persian transcripts were re-read by Persian speakers during these sessions where the translations were not sufficiently clear. This process helped to validate the thematic codes, reduce confirmation bias, increase internal validity of findings, and update the coding framework. Internal validity was further increased by triangulation of interview results with information gathered from the literature review.

## Results

###  Iranian History, Politics, and Religion Shape the Contours of its Civil Society and Civic Engagement

 Iran’s long history of civic engagement and philanthropy is enshrined in its culture, religious thought, and also in social spheres such as health. The term ‘civil society’ was essentially imported and became increasingly used to denote civic activity in the late 1990s, but its definition in the Iranian context has never been completely clear.^12,13^ Our literature review helped confirm, however, that the ideals represented by international development notions of civil society and civic engagement have been present in the Iranian psyche for centuries and “have been influential in shaping social, political, and economic life.”^12^

 A case in point are religious charities, characterized as a foundational element of Iran’s civil society, and urban NGOs. Their valued social services (aid to poor children and orphans, for example) are a mainstay of participatory engagement at local levels in some areas. An estimated 14 000 to 26 000 charities, NGOs, community funds or foundations exist in the country, with 10% of them working in the health sector (Directorate-General for NGOs and Community Organizations, Ministry of Health and Medical Education, Written communication, January 23, 2018).^14^ Charity is also a large focus for Iran’s 93 000 religious entities.^15^ Given these large numbers, volunteer and charity work is at the core of Iranian community life, a vehicle for social participation of certain sectors of the population.

 We draw on Hegel’s view of civil society as a product of history to better understand organized forms of civic engagement in the Iranian health sector.^16^ This implies viewing civil society in Iran as the collective internalization of a civic sense as well as the civic activity stimulated by it. Put this way, it is clear that individual charitable action and community support for the poor has always been part and parcel of the population’s fabric.^12^

 The concept of civil society used in the international development world is, however, founded on the idea of the state and civil society being two separate entities, with civil society being explicitly ‘non-state’ in character, as an either opposing or complementary force to the state, depending on the context. However, if civil society is rather a product of a people’s history, in Iran the state has “historically stood at the top of society as a paternalistic figure with responsibility for welfare,”^12^ ie, disentangling the blurred boundaries between the state and the people would mean missing the majority of participatory activities – especially in social sectors such as health where welfare and charitable work often see the state and non-state actors collaborating in tandem.^17^

 Due to government changes and a volatile political context, there is also an evolving character to how civil society and civic action is regarded and manifested in Iran; in this context, considering civil society as a dynamic process makes more sense, rather than branding it as a static entity with definitive structures. With this in mind, we highlight in the next sections what the different forms of civil society mean in Iranian policy and practice.

###  Unpacking Organized Forms of Civic Engagement in the Iranian Context (See Box 1 )

 The official definition of ‘NGO’ as expressed by a MoHME key informant was *“an organization that is legal, non-profitable, independent and voluntary. It supports the well-being of the people, especially the disadvantaged class. This is the definition that we’ve added in the Ministry of Health, in particular the disadvantaged class.*”^18^ The interviewee went on to specify that “*charity or charity enterprises are more well-off people who want to do charity work, their work is more financial assistance. For example, they give cash to orphans or widowed women. The NGOs that we recognize as the NGO do the scientific work*.*”*^18^

**Box 1. Unpacking Organized Forms of Civic Engagement Within the Iranian Context**

The term **‘charity’** is used more for *financing* charitable works whereas the term **‘NGO’** has the connotation of charitable *action*.

**CBOs** conduct grassroots work in health, with close ties to communities, and a focus on the poor and underprivileged.

**Informal and de-facto formal groups** are often grassroots in nature and may be linked to state and para-statal institutions.

--------------

Abbreviations: NGOs, non-governmental organizations; CBOs, community-based organizations.

This explanation indicates that NGOs are non-profit entities with no paid staff, mainly engaging in technical work and service delivery, and independent of the government or any political or religious agenda. While several NGOs do fit this definition, in practice many do not – for example, many NGOs do have paid positions and/or are linked to government entities. Nevertheless, this definition helps greatly in understanding most NGOs are *most likely *engaging in, which many study interviewees confirmed was largely curative care and patient support linked to specific diseases, and how they tend to operate.

 The insight provided by the above quote on charities can be best understood when considering the Iranian (and Islamic) tradition of giving to the underprivileged. Since the focus of the term ‘charity’ is on financial assistance more than anything else, NGOs are often seen as charities if they also undertake fundraising and have wealthy donors. In this case, the same institution can thus be functionally both an NGO as well as a charity. As the Chief Executive Officer of a reputed cancer charity hospital confirmed in his interview, “*25 years ago charity organizations were registered under Article 10 of the Law of the Parties in Iran…such as [ours]…but in essence and unofficially, all recognize [us] as an NGO in Iran.”*^19^

 CBOs have a long and active tradition in social sectors such as health, without necessarily being labelled as such. Indeed, volunteer work is ingrained in Iranian community life, with a high level of population willingness to contribute to their communities. CBO work is traditionally localized and grassroots in nature, rather informal in some places but formalized in others, and not traditionally under the direct control of the state nor private sector. One interviewee characterized CBOs as *“the association…that is formed by the local residents with a local identity. Its difference with an NGO is that it does not have bureaucracies of registration and is formed based on an identity.”*^20^

 That is, however, only partly the case: during the reform movement of the 1990s, some CBOs were increasingly linked to or merged with state-sponsored health programmes (bringing with it bureaucratic procedures) due to the synergies and complementarities they offered, besides low-cost health programme delivery. To complicate matters further, many grassroots activities are often done in collaboration with institutions with close ties to state or parastatal entities, such as mosques, which inevitably lends itself to closer merging with government health activities.

 As insinuated by the above quote, grassroots activities in health are also conducted by numerous informal social groups who are not registered with any government body but may be better organized and impactful than formal CBOs. As an MoHME interviewee affirmed, *“A large number of organizations and social groups are into charity work, hundreds of thousands, but they do not have legal status.”*^21^ This may be linked to a wish to stay as independent as possible from government intervention or religious convictions to stay anonymous while giving.^22^

 Like CBOs, the focus of many of these informal social groups is serving the poor and underprivileged; they too have close ties to local communities and are heavily dependent on community networks. Many of them use the infrastructure of formal clerical organizations^12^ but remain informal. Others have been merged with organized health activities under the patronage of the Supreme Leader, making them de facto formal. Indeed, the latter have vast resources and capacity, as well as trust and familiarity of communities as a basis of their support and influence. The same applies for the work of other quasi-civil society institutions such as the social services branch of the Basij paramilitary organization which sometimes engages in health promotion and prevention, as well as curative care.

###  A Closer Look at Government-Led Participatory Governance Initiatives

 The 1990s saw the Iranian government actively fostering participation in health for health programmes such as primary healthcare or women’s health^[1]^, in part as an (effective) cost minimization strategy. The central government designed the programmes with participation as a key component of programme implementation. Our literature review revealed a multitude of studies on local, community-based health initiatives, mainly funded by the central government, with programme delivery undertaken largely by communities and volunteers (some, as mentioned previously, labelled as ‘NGOs’).^23-25^ However, the community volunteers were mainly given implementation tasks, with monitoring and decision-making remaining within government circles. Several studies pointed to the top-down nature of programme management,^24,26,27^ with one study concluding that “according to the participants (of community-based health programmes), governmental programmes have centralised decision-making and management processes and local volunteers have no role in selecting managers at different levels of a programme.”^24^

 In the early 2000s, however, the Tehran Municipality began leveraging citizen participation in health for both programme implementation *and* decision-making.^28^ A Municipality-run Neighbourhood Health House (labelled as a CBO) staff member emphasized this point: *“[T]he Tehran municipality, after years of taking care of the affairs by itself, dared to entrust the management of the affairs to the people. [Then]…this structure took shape and was sustained and the municipality…assumed the supportive role to help the people.”*^29^ Our qualitative data demonstrated that municipal health planners duly examine feedback from programme volunteers relaying concerns of the community, thereby ensuring responsive health planning. One volunteer summed it up: “*In principle we transfer the feedback of the community to [the municipality]. They get more familiar with the problems and demands of the people.”*^29^

 The HTP’s push to ‘socialize’ all health activities lead to the creation of the MoHME Deputy Ministry of Social Affairs in 2016 whose explicit mission was to boost people’s participation in health policies and programmes. This led to the first series of Provincial Health Assemblies as well as the first National Health Assembly (NHA) in 2017. In February 2019, the Deputy Ministry’s responsibilities were integrated into other MoHME deputies’ activities or directly transferred to the Minister’s Office.^30^

 With the provision of additional financial resources through the HTP, many existing participatory governance mechanisms which were not always fully functional or effective were further strengthened. Table shows a non-exhaustive list of such mechanisms. Yet municipality health houses, health clubs, rural health houses, health posts, health centres and people’s participation houses all have some level of overlapping jurisdiction and service duplication – this may or may not be responding to a true community need as the different institutions have arisen in a specific historical context.

**Table:** Facilities for Public Participation in Health in Iran

**Facility Name**	**Description**
Municipality health houses and health club	In cities (mainly Tehran), each district has a health house which organizes clubs on health topics (eg, diabetic club, elderly health club, blood transfusion club, etc). These clubs serve to educate the public and patients on its health topic of focus. Some also provide consultation and counselling, under the supervision of the municipality’s Director of Health. All services are provided by volunteers.
Rural health houses	*Rural health houses* are primary care facilities under the supervision of MoHME. Rural health houses are mainly run by *behvarz* who are selected from the same village and trained in basic health services by the government. Some rural health houses have additional volunteer staff who support the *behvarz* in their service delivery and outreach work.
Urban health post	Same as rural health houses but located in urban areas.
Comprehensive health centre (urban and rural)	*Comprehensive health centres* have trained, government-employed professional medical staff to provide the second level of service delivery under the supervision of MoHME. Comprehensive health centre staff also supervise the health houses and health posts, and are thereby involved in participation-related activities.
People’s participation house	*People’s participation houses* are essentially CBOs governed and run by 21 representatives from different constituencies, including for example teachers, retired persons, currently active workers, basij, religious groups, etc. This governing body brokers between decision-makers and the population whom they cover within their catchment area. They are unpaid volunteers.

Abbreviations: MoHME, Ministry of Health and Medical Education; CBOs, community-based organizations. Source: adapted from Rajan et al.^31^

## Discussion

###  Overlapping Concepts Reflect the Reality of Duplication and Fragmentation of Participatory Activities

 Ensuring a minimum level of coordination between the somewhat disparate formal, de facto formal, and informal participatory activities could bring more coherence in terms of strengthening the culture of ‘socialization’ in service of HTP goals. The coordination is necessary amongst the government-led projects and programmes – our qualitative analysis demonstrated that they sometimes overlap with or duplicate each other. Besides missing out on cross-learning across the various pilot programmes, municipality initiatives, and project-based research work, the competition it creates is contrary to HTP ambitions. For example, municipality-led and centrally-led initiatives at times use the same volunteers to do similar activities.

 The urgent need for government to take on a major coordination role applies also for activities which are carried out by civil society, quasi-state organizations, or others, in order to align them towards HTP objectives. Coordinating activities is not equal to controlling the activities, but rather harnessing the willingness of stakeholders to contribute to the HTP. In terms of the participatory governance mechanisms available to the public (see Table), a useful exercise would be to specifically examine how municipal and centrally-funded services, including their respective approaches to participation, could explicitly complement and cross-learn from each other’s experiences.

 Coordinating targeted efforts to engage with the population for health must go hand in hand with the culture change envisioned in the MoHME commitment to ‘socialize’ the way the health sector works. That culture change is still needed as many programmes and health initiatives are still run in a top-down way. Participatory decision-making is not yet a widespread phenomenon. A coordinated, holistic, and common approach to eliciting and encouraging participation can help channel efforts towards common public health goals as spelled out in the HTP.

###  Invest in Government Capacities to “Do” Participation

 Apart from national-level commitment and coordinating efforts, a focus on influencing the mindset and ‘modus operandi’ of government officials in the long-term is needed. Interviewees stressed that some government entities not only lack confidence in the ability and utility of civil society, but often view them as direct competitors instead of partners. One provincial representative summed it up flatly: *“Our authorities.. think we are going to take the position from them.”*^32^ This highlights the urgent need for capacity building for mid-level cadres to better understand the added value of engagement with civil society, communities and the public, ultimately to support their own policy work and rendering it more responsive to the needs of the Iranian people, a true ‘win-win’ opportunity on all sides.^33^ Government skills-building for participatory processes would also help make health programme management less top-down, as noted in our findings.

 Overall, the HTP reforms have led to more government engagement with civil society, for example with one group of actors (CBO and NGO actors), as underlined by a parliamentarian key informant: *“Certainly … the MOH … they identify the CBOs, organize them, and direct them to where it is needed, [it] is definitely effective, and I think it was a positive work that, fortunately, [was] undertaken and should be strengthened.”*^34^ For other important non-state or quasi-state actors in health, more efforts could be made to bring them into discussions. At the very least, an exchange of information with such actors could avoid expensive duplication of activities and resource waste. A promising avenue for intensified engagement and coordination are the local, provincial, and national health assemblies as a platform for engagement.

###  The NHA as a Potential Opportunity to De-fragment the Approach to Meaningful Participation 

 The NHA concept has been successfully tried elsewhere^35^ and has demonstrated its potential to bring together stakeholders, including the population, to examine, discuss, and find viable solutions for health sector challenges, while simultaneously drawing on the same stakeholder base to help implement those very solutions.

 Iran’s First NHA took place in 2017^36^ which triggered a series of provincial and local health assemblies shortly thereafter. So far, the NHA has remained a one-off exercise due to shifting political priorities, underlining the challenge of sustaining participatory engagement models, coupled with a missing legal framework that could serve as a means to ensure long-term commitment. The NHA experience in Iran was however positively recognized by many actors as a useful platform for engaging and coordinating amongst actors which could be picked up in the future again.

 Subsequent NHAs could bring together municipality staff working on health as well as central Ministry authorities, semi-governmental organization health programme volunteers, religious charities, scientific associations, research centres, trade unions, representatives from other sectors, representatives from judiciary organs etc. to exchange on their respective health-related activities, thereby assisting to reduce duplication and fragmentation. Collaboration and coordination where it has not existed before will not necessarily be easy; however, a platform such as the NHA could facilitate this greatly by providing an official regular event where exchange and debate can take place. The strength of the platform will be dependent on ensuring that all decisions taken via this platform are official, enforced and implemented.

 The NHA can also serve as a potential channel for NGOs and charities to influence national-level health decision-making, thereby better connecting the local with the national. To date, much of the long-term local participatory programmes have remained local in nature – those that are centrally-funded tend to be one-off pilot projects which have not always taken hold as long-term institutions (with some notable exceptions). It would be an immense missed opportunity if the different needs, views, and willingness to contribute embodied in the multitude of local participatory health activities were not adequately channelled towards sustainable health goals as outlined in the HTP.

###  Lessons for Other Country Settings

 The Iranian experience provides valuable lessons learnt for other countries who are in the process of setting up, strengthening and institutionalizing public participation models for improved health policy-making. Our findings confirm that the path to meaningful participatory governance as a health sector modus operandi is not straightforward, but rather non-linear, with significant ups and downs affected by changing socio-economic contexts and government priorities. Other middle-income countries, such as Brazil and Mexico, have experienced, and are still experiencing, similar upheavals through periods of reform and policy struggles.^37,38^ A key lesson here is the need to persevere through the ups and downs, as building a lasting culture of social participation is no short-term affair. It takes time, effort, and learning to develop into a system which works for the local context; this might require adapting international conceptualizations of interaction between the state and its people to different political or regional settings.

 Institutionalizing people’s voice and civil society participation also requires a longer-term vision which works towards bringing together and linking participatory activities happening at various system levels and within different health programmes. For Iran, this could be the NHA mechanism. In other middle-income countries, policy researchers have proposed similar set-ups, such as a ‘Citizen Observatory’ in Mexico.^37^ In Brazil, the National Conferences on Health – with 50% of participants being health service users – consolidate deliberations at the municipal, state, or regional levels as well as those within decentralized Health Councils.^39^ These conferences are recognized as a national public good to amalgamate people’s voices emanating from different platforms to feed into more responsive and implementable health policies.^40^ The acknowledgment of such mechanisms as public goods was also affirmed in a study of Thailand’s well-established NHA mechanism.^35^

 Like Iran, many countries’ government cadres require a considerable capacity boost to be able to competently initiate, manage, and sustain participatory processes in health. Most civil servants working in health were recruited for their more traditional biomedical and technical skill sets; the abilities needed to meaningfully engage with people from all walks of life, with those with opposing views, and with those whose vested interests may not be completely in line with the public one, is challenging to say the least, especially when that engagement needs to be carefully channelled towards policy-relevant outcomes. It requires immense practice, mentoring, and a bureaucracy which explicitly values both the process and outcome. Political prioritization at the highest levels is thus needed, along with investment in mid-level government cadre capacities to practically undertake participatory activities.^33^ Even well-reflected, mature processes such as Thailand’s NHA require embedded capacity-building and awareness-raising initiatives to ensure policy relevance and sustainability.^41^

 In conclusion, our study demonstrates that including participatory governance into the design of UHC reforms needs to begin with a more localized understanding of civil society and civic space. Key design elements also include embedded capacity-building of government cadres and mechanisms to coordinate and consolidate existing participatory initiatives into a common forum to learn from and exchange with each other.

## Acknowledgements

 We acknowledge research support from Csongor Bajnoczki.

## Ethical issues

 WHO undertook this work within its programmatic mandate and hence did not go to an Ethics Review Board.

## Competing interests

 Authors declare that they have no competing interests.

## Authors’ contributions

 DR conceptualized the study design. DR and KK drafted the manuscript. DR, NR, MM-L, and MR undertook data acquisition and analysis, and with MHA, BD, AAF, RM provided valuable inputs to the manuscript. All authors have approved the final manuscript.

## Funding

 WHO and Iran’s Ministry of Health and Medical Education (MoHME) co-funded the overall evaluation.

## Endnote

 [1] Central government-led initiatives such as the Volunteer Health Worker programme is one such programme, which began in 1992 as a small project with 200 women, mainly from low-income neighbourhoods in Tehran; by 2007 the official numbers approached 100 000 women.
